# Strategy to Better Select HIV-Infected Individuals for Latent TB Treatment in BCG-Vaccinated Population

**DOI:** 10.1371/journal.pone.0073069

**Published:** 2013-08-27

**Authors:** Chin-Hui Yang, Pei-Chun Chan, Say-Tsung Liao, Shu-Hsing Cheng, Wing-Wai Wong, Li-Min Huang, Po-Ren Hsueh, Hung-Yi Chiou

**Affiliations:** 1 Centers for Disease Control, Department of Health, Taipei, Taiwan; 2 Ph.D. Program, School of Public Health, Taipei Medical University, Taipei, Taiwan; 3 Division of Infectious Diseases, Department of Internal Medicine, Taipei Medical University Hospital, Taipei Medical University, Taipei, Taiwan; 4 Division of Rheumatology, Department of Internal Medicine, Taipei Medical University Hospital, Taipei Medical University, Taipei, Taiwan; 5 Division of Infectious Diseases, Department of Internal Medicine, Taoyuan General Hospital, Taoyuan, Taiwan; 6 Division of Infectious Diseases, Department of Medicine, Taipei Veterans General Hospital, Taipei, Taiwan; 7 Departments of Pediatrics, National Taiwan University Hospital, National Taiwan University College of Medicine, Taipei, Taiwan; 8 Departments of Laboratory Medicine and Internal Medicine, National Taiwan University Hospital, National Taiwan University College of Medicine, Taipei, Taiwan; 9 School of Public Health, Taipei Medical University, Taipei, Taiwan; University of Padova, Medical School, Italy

## Abstract

**Objective:**

To evaluate the T-SPOT.TB interferon-γ releasing assay and the tuberculin skin test (TST), for the diagnosis of latent tuberculosis infection(LTBI) and the development of subsequent active tuberculosis, in BCG-vaccinated HIV-infected individuals.

**Methods:**

HIV-infected individuals without clinical suspicion of active TB or a past history of TB were enrolled from 1 January 2008 to 30 November 2010. Both T-SPOT.TB test and TST were offered to the participants whom were followed up prospectively until April 30, 2012 for development of TB.

**Results:**

Among the 909 participants, 25% had positive TST reactions with cut-off point of 5 mm and 15% had positive T-SPOT.TB results. After a median follow-up of 2.97 years, there were 5 cases developed culture-confirmed active TB (all had dual positive TST and T-SPOT.TB results), and the incidence was 0.17 per 100 person-years. The relative risks (RRs) for subsequent active TB in HIV-infected individuals with positive TST results, positive T-SPOT.TB results and dual positive results compared with the risk for individuals with negative results were 40.6 (95% CI 2.1–767.9), 73.9 (95% CI 3.9–1397.7) and 226.5 (95% CI 12.0–4284), respectively. The number needed to treat to prevent one subsequent TB case among patients with a positive TST, a positive T-SPOT.TB and dual positive results was 35, 22 and 8 respectively.

**Conclusions:**

Adopting positive results of the TST and T-SPOT.TB to screen LTBI among BCG-vaccinated HIV-infected individuals might be feasible. Number needed to treat for isoniazid preventive therapy could be reduced significantly by using dual positive strategy.

## Introduction

HIV-infected individuals have a higher rate of progression from latent tuberculosis infection (LTBI) to active TB than do non-HIV-infected persons, even with the use of effective antiretroviral therapy and the relative risk reached ten-fold [1], [2], [3], [4]. Isoniazid preventive therapy (IPT) reduced the risk of TB activation in HIV-infected TST-positive LTBI subjects by 40% to 76% [5], [6], [7], [8]. There were around 20,000 people living with HIV(PLHIV) in Taiwan by the end of 2012, the major transmission categories were male-to-male sexual contact, injection drug use and heterosexual contact [9]. Though free highly active antiretroviral therapy policy reduced mortality rate and the incidence of opportunistic infections in HIV-infected persons, TB along with *Pneumocystis carinii* pneumonia remained the most common opportunistic infections in PLHIV in Taiwan [10]. Through the implementation of directly observed treatment strategy in 2006, the TB incidence in Taiwan decreased gradually from 73/10^5^ in 2005 to 57/10^5^ in 2010 [11]. With decreasing new TB case number, diagnosis and treatment of LTBI in high-risk populations has become the next important strategy in accelerating TB elimination.

The tuberculosis skin test (TST) is the traditional method for the diagnosis of LTBI, but the test has low specificity due to cross reactions with the bacillus Calmette-Guérin (BCG) vaccine and non-tuberculous mycobacteria. Taiwan launched the neonatal BCG vaccination since 1965 and the coverage rate scaled up rapidly to over 80% since 1970 and continued above 97% after 2001 [12]. BCG booster program for schoolchildren was stopped since 1997, thus people born before1986 could receive two or more BCG vaccination. The high BCG vaccination coverage rate and booster policy hampered the use of TST to diagnose LTBI on those who were born before 1986 [13]. In addition, TST has low sensitivity in HIV-infected patients due to anergy, particularly in patients with advanced immunosuppression [14]–[15]. Thus, the detection and treatment of LTBI to prevent progression to active TB disease are crucial for controlling HIV-associated TB, yet the diagnosis tool for LTBI is still debated.

A method based on the measurement of interferon-γ release by T cells incubated ex vivo with *Mycobacterium tuberculosis* antigens was developed recently and addresses many of the TST's limitations. One meta-analysis suggested that interferon-γ release assays (IGRAs) had higher specificity for TB infection than the TST does, especially in BCG-vaccinated, non-HIV infected populations [16]. Only a few studies found that IGRA had higher sensitivity among immune-compromised hosts including HIV-infected individuals [17], [18], [19]. Thus the use of IGRA, compared to TST, as LTBI screening in BCG-vaccinated, HIV-infected individuals becomes an important issue in countries with moderate TB burden.

The objectives of this prospective, longitudinal study were to evaluate the T-SPOT.TB (an IGRA) and the TST, for the diagnosis of LTBI and the development of subsequent active tuberculosis, in BCG-vaccinated HIV-infected individuals. We tried to determine the clinical and epidemiological risk factors associated with positive results for the TST and the T-SPOT.TB test in HIV-infected individuals and recommend an appropriate protocol to better select HIV-infected individuals for IPT.

## Patients and Methods

### Study Settings

Participants were recruited on a voluntary basis from three HIV outpatient clinics (Taipei Medical University Hospital, Taipei City Hospital, and Taoyuan General Hospital) and two prisons (Taipei Prison and Taoyuan Prison) between January 1, 2008 and November 30, 2010. There was a total of 1000∼1200 HIV-infected patients followed up in the three HIV clinics regularly. Taipei prison accommodated 3,000 adult male inmates and around 200 among them were HIV-infected. Taoyuan prison accommodated 1,000 adult female inmates and around 150 among them were HIV-infected. According to Taiwan's regulation, inmates have been mandatorily tested for HIV upon entry to correctional facilities since 1990 [20]. HIV/AIDS infected individuals are provided with free medical care, including anti-retroviral treatment, by the government of Taiwan since 1997. Inmates were also covered with free HIV-related care. According to the national guideline, HIV-infected individuals with CD4+ lymphocyte count <350 cells/mm^3^ are recommed to initiate antiretroviral therapy [21]. The two prisons were contracted with Taipei Medical University Hospital and Taoyuan General Hospital to offer routine HIV-related care.

### Ethics

The study was approved by the Centers for Disease Control, Department of Health, Executive Yuan, Taiwan; the Institutional Review Board (IRB) of Taipei Medical University Hospital and the IRBs at Taipei City Hospital and Taoyuan General Hospital. All participants gave written informed consent.

### Eligibility

Individuals aged 18 years and above and had HIV infection (confirmed by Western blot tests) were enrolled. Individuals with active TB or past history of TB were excluded. Written informed consents were provided to all participants before receiving screening tests. Individuals who understood the screening programme and who gave informed consent were enrolled. All potential participants who declined to participate or otherwise did not participate were eligible for treatment (if applicable) and were not disadvantaged in any other way by not participating in the study.

### Demographic data collection

Each patient's HIV-related information was collected by a review of medical records and from the Taiwan CDC HIV registry data. After consent was obtained, the standardized questionnaire was completed by all participants which including background sociodemographic and clinical information. The study nurse checked the presence of BCG scars prior to the examinations provided. The CD4+ lymphocyte count and HIV RNA load data were collected.

### Evaluation Procedures

Baseline chest radiographs for each participant and sputum cultures for *M. tuberculosis* collected for symptomatic patients were used to exclude active TB. The TST with purified protein derivative (RT 23 2 TU, Statens Serum Institute, Copenhagen, Denmark) and the T-SPOT.TB test (Oxford Immunotec, Oxford, United Kingdom) were done with the flow of drawing blood first followed by TST. The blood was processed by laboratory staff blind to the clinical status of the sample and interpreted according to the manufacturer's protocol. The transverse induration size of the TST was read by the nurses who administered the test between 48 and 72 hours after administration, induration size exceeding 5 mm was considered positive [22]. IPT was offered to recruited participants with positive TST reactions with free isoniazid, 300 mg daily for 9 months [23].

### Surveillance for active TB disease during the follow-up

All patients, including those who declined treatment, were instructed on symptom-specific self-monitoring and reporting. Chest radiographs and sputum cultures for *M. tuberculosis* were routinely performed for symptomatic patients [21]. All enrolled subjects were routinely followed up every 3–6 months in the HIV clinics until April 30, 2012; death; or the development of active TB, whichever was earliest. Cross-matching with the national TB and death registries was also performed at the end of the follow-up to allow data capture for any cases lost to follow-up. An active case of TB was defined as disease proven by the isolation of *M. tuberculosis* from sputum or other tissue.

### Performance of both tests and numbers needed to treat with IPT

We evaluated the performance of TST and T-SPOT.TB with regard to the development of active TB in participants who did not receive IPT. Assuming a full protection of IPT, we will further calculate the number needed to treat to prevent subsequent TB disease for each test.

### Data Analysis

Multivariate logistic regression was used for cross-sectional analysis with adjustment for all relevant potential confounders associated with positive TST, T-SPOT.TB and dual positive results. The concordance between the TST and T-SPOT.TB results was assessed using Cohen's kappa (κ) coefficient. The strength of the agreement was considered ‘poor’ for ≤0.20, ‘fair’ for 0.20<κ≤0.40, ‘moderate’ for 0.40<κ≤0.60, ‘substantial’ for 0.60<κ≤0.80 and ‘optimal’ for 0.80<κ≤1.00 [24].

One main outcome of interest was the incidence rate of active TB, stratified according to screening test and IPT use. To calculate the relative risks (RRs) of rare events, we used the Poisson distribution in survival analysis. The Kaplan-Meier survival analysis with a log-rank test was conducted for analysis of the effect of IPT. All statistical analyses were two-sided and were considered significant when the *P* values were less than 0.05. Statistical analysis was performed with using SAS, version 9.2 software (SAS Institute, Cary, North Carolina, USA) and SPSS v 14.0 (SPSS Inc, Chicago, Illinois, USA).

## Results

### Characteristics and test results of the study participants

**Figure 1:**
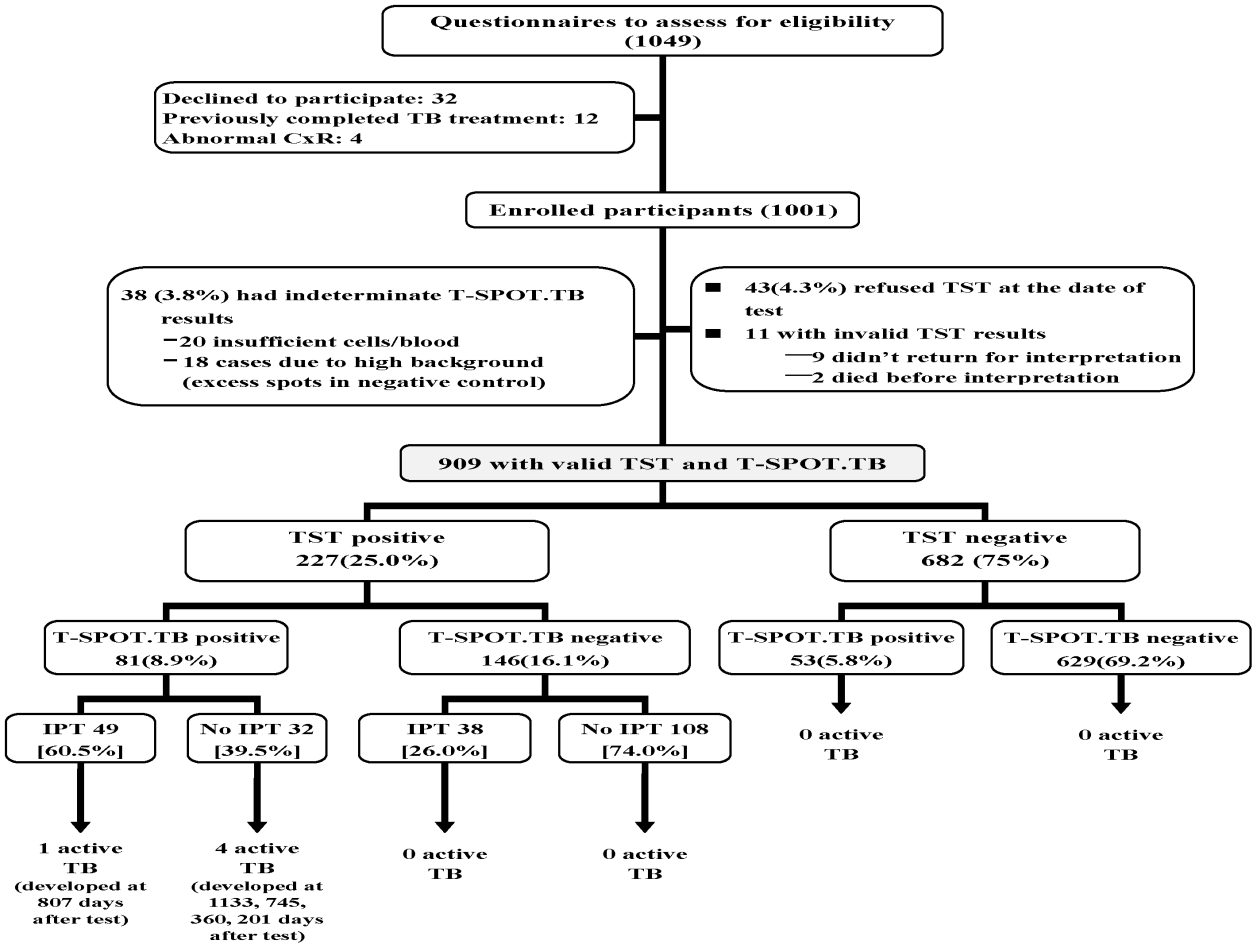
Flow chart showing 909 HIV-infected individuals recruited for final analysis. T-SPOT.*TB*  = (commercial form of interferon-gamma release assay from Oxford Immunotec, Abingdon, UK); TST =  tuberculin skin test; IPT = isoniazid preventive therapy.

A total of 1,049 HIV-infected individuals were invited to complete the questionnaires for enrolment (Figure 1). The TST completion rate was 94.6% and majority of the patients (62.6%) had an anergic TST reaction (TST = 0 mm) regardless of the result of the T-SPOT.TB test. There were 25% of the participants had positive TST results (TST≥5 mm), with a median induration of 11 mm (≥10 mm, 52.5%; ≥15 mm, 19.5%). Patients with positive T-SPOT.TB results tended to have bigger TST size compared to those with negative results (12.5 mm vs. 7.8 mm, p<0.001).

For the T-SPOT.TB test, 96.2% of the enrollees had a valid result, and 15% of them had positive results; the 38 patients (3.8%) with indeterminate results were similar to those with valid results in terms of TB risk factors and demographics.

A total of 909 individuals had interpretable results for both the T-SPOT.TB test and the TST and were used for further analysis. Background characteristics of enrollees are summarized in Table 1. The distribution of HIV transmission risk categories among enrollees was similar to that for the national registry data.

**Table 1:** Demographic data of enrolled participants at time of simultaneous T-SPOT.TB and TST.

	All patients (n = 909)	TST positive (n = 227, 25.0%)	T-SPOT.TB positive (n = 134, 14.7%)	Dual positive (TST and T-SPOT.TB) (n = 81, 8.9%)
**Age, median(yrs)**	34.8(20–82)	34.8(21–82)	37.4(21–82)	36.7(21–82)
**Men**	796(87.6%)	198(87.2%)	122(91.0%)	75(92.6%)
**HIV transmission risk category**				
**MSM**	450(49.5%)	84(37.0%)	49(36.6%)	29(35.8%)
**Heterosexual**	97(10.7%)	28(12.3%)	17(12.7%)	10(12.3%)
**IDU**	362(39.8%)	115(50.7%)	68(50.7%)	42(51.9%)
**HIV-related factors**				
**AIDS status**	196(21.6%)	34(15.0%)	25(18.7%)	17(21.0%)
**CD4 cells, median**	447(7–1644)	547(91–1460)	491(124–1644)	524(124–1460)
**CD4<200/ml**	56(6.2%)	5(2.2%)	6(4.5%)	4(4.9%)
**HIV RNA, median(log10)**	3.25(1.4–6.5)	2.85(1.4–5.4)	3.08(1.4–5.4)	3.38(1.4–5.4)
**HIV RNA<50 copies/mL**	304(33.4%)	87(38.3%)	48(35.8%)	27(33.3%)
**On ART at enrollment**	369(40.6%)	90(39.6%)	53(39.6%)	32(39.5%)
**No BCG scar** #	51(5.8%)	11(4.9%)	15(11.2%)	8(9.9%)
**Self-reported contact with active TB case** #	52(6.3%)	19(9.4%)	15(11.8%)	13(17.3%)
**Prison stay**	362(37.9%)	121(53.3%)	66(49.3%)	42(51.9%)

*Definition of abbreviations*: MSM = man who had sex with man; IDU = injecting drug use; ART = highly active antiretroviral therapy; BCG = Bacille Calmette-Guérin; TST =  tuberculin skin test.

# 83 cases missed data of BCG scars; 133 cases missed TB contact history.

### Predictive factors for positive TST and T-SPOT.TB results

Table 2 shows the independent predictors for positive TST and T-SPOT.TB results in the multivariate logistic regression analysis. Increasing CD4+ lymphocyte counts and a history of contact with TB case were independently associated with a positive response in both tests. Increasing age and male gender independently predicted a positive T-SPOT.TB response, but not TST. Prison stay for over 6 months was associated with positive TST response, but not with positive T-SPOT.TB response. Further analysis found the proportion of two BCG scars was higher among participants with prison stay than the other participants, 44.5% vs. 38.6%, *p* = 0.08. Repeat the analysis with or without prison stay revealed similar results for other covariates in the model.

**Table 2:** Predictors of positive TST and T-SPOT.TB result in multivariate logistic regression analysis.

	Positive TST			Positive T-SPOT.TB			Dual Positive*		
Variables	aOR	CI	*p* value	aOR	CI	*p* value	aOR	CI	*p* value
Age	0.99	0.97–1.01	0.444	**1.02**	**1.00–1.05**	**0.034**	1.01	0.99–1.04	0.345
Male gender	1.46	0.87–2.47	0.157	**2.34**	**1.17–4.68**	**0.016**	**3.33**	**1.27–8.76**	**0.015**
HIV transmission risk category									
MSM	1			1			1		
Heterosexual	1.74	0.97–3.12	0.065	1.63	0.84–3.17	0.147	1.91	0.80–4.55	0.144
IDU	1.08	0.53–2.18	0.830	1.97	0.88–4.42	0.101	1.63	0.54–4.87	0.385
HIV-related factors									
AIDS status	1.09	0.65–1.82	0.756	1.14	0.63–2.06	0.675	1.69	0.78–3.66	0.185
CD4+ lymphocyte (per 100 cells/mm^3^)	**1.25**	**1.16–1.36**	**0.000**	**1.16**	**1.06–1.26**	**0.001**	**1.31**	**1.17–1.48**	**0.000**
HIV RNA(log10)	**0.78**	**0.63–0.97**	**0.023**	0.96	0.75–1.22	0.753	1.04	0.75–1.44	0.800
On ART at enrollment	0.78	0.39–1.57	0.493	0.96	0.43–2.13	0.921	1.15	0.39–3.41	0.799
BCG vaccination	1.21	0.57–2.57	0.617	0.51	0.25–1.02	0.058	0.58	0.23–1.45	0.246
Self-reported contact with active TB case	**2.10**	**1.10–4.01**	**0.024**	**2.77**	**1.42–5.42**	**0.003**	**4.11**	**1.90–8.89**	**0.000**
Prison stay#	**2.67**	**1.34–5.31**	**0.005**	1.31	0.59–2.88	0.510	2.41	0.81–7.11	0.112

*Definition of abbreviations*: aOR = adjusted Odds ratio; CI = 95% confidence interval; MSM = man who had sex with man; IDU = injecting drug use; ART = highly active antiretroviral therapy; BCG = Bacille Calmette-Guérin; TST =  tuberculin skin test.

*use participants with dual negative results as the reference group.

# duration over 6 months.

All variables were included in multiple logistic regression analysis for adjustment.

### Agreement between the TST and T-SPOT.TB results

Table 3 summarizes the agreement between the T-SPOT.TB and TST results stratified by age, CD4+ lymphocyte count group and BCG vaccination status. Except in the participants with CD4+ count <200/mm^3^ or without BCG vaccination, TST had significantly more positive results compared with T-SPOT.TB. The overall agreement between the two tests was only fair (78.1%, κ = 0.32). The agreement and kappa values between the two tests was highest in individuals with CD4 cell counts <200 cells/mm^3^ (94.6%, κ = 0.70), followed by individuals who had not received the BCG vaccine (80.4%, κ = 0.49) and individuals aged 40 years or above (80.1%, κ = 0.41).

**Table 3:** Agreement between T-SPOT.TB assay and Tuberculin Skin Tests, stratified by age and CD4+ lymphocyte counts groups and BCG vaccination status.

		TST*	T-SPOT.TB	TST and T-SPOT.TB	Agree-ment	Kappa (95% CI)
		(% of the category)				
**All patients**	(n = 909)	25.0%	14.7%	8.9%	78.1%	0.32(0.24–0.41)
**Age**						
<30 years	(n = 221)	21.7%	9.5%	5.4%	79.6%	0.25(0.05–0.44)
30–39 years	(n = 407)	28.3%	13.5%	8.8%	75.9%	0.30(0.17–0.41)
≧40 years	(n = 281)	22.8%	20.6%	11.7%	80.1%	0.41(0.28–0.55)
**CD4+ lymphocyte count**						
<200	(n = 56)	8.9%	10.7%	7.1%	94.6%	0.70(0.37–1.00)
200–349	(n = 225)	14.7%	8.0%	4.0%	85.3%	0.28(0.05–0.51)
350–499	(n = 265)	23.8%	17.4%	9.1%	77.0%	0.30(0.150.45)
≧500	(n = 363)	34.7%	17.6%	12.1%	71.9%	0.30(0.18–0.42)
**BCG scar**						
0	(n = 51)	21.6%	29.4%	15.7%	80.4%	0.49(0.20–0.77)
1	(n = 464)	24.6%	15.1%	9.5%	79.3%	0.36(0.24–0.47)
2	(n = 358)	27.4%	13.7%	8.1%	75.1%	0.26(0.13–0.39)

*Definition of abbreviations*: BCG = Bacille Calmette-Guérin; TST =  tuberculin skin test.

*A TST reaction ≧5 mm was considered as positive.

**Figure 2:**
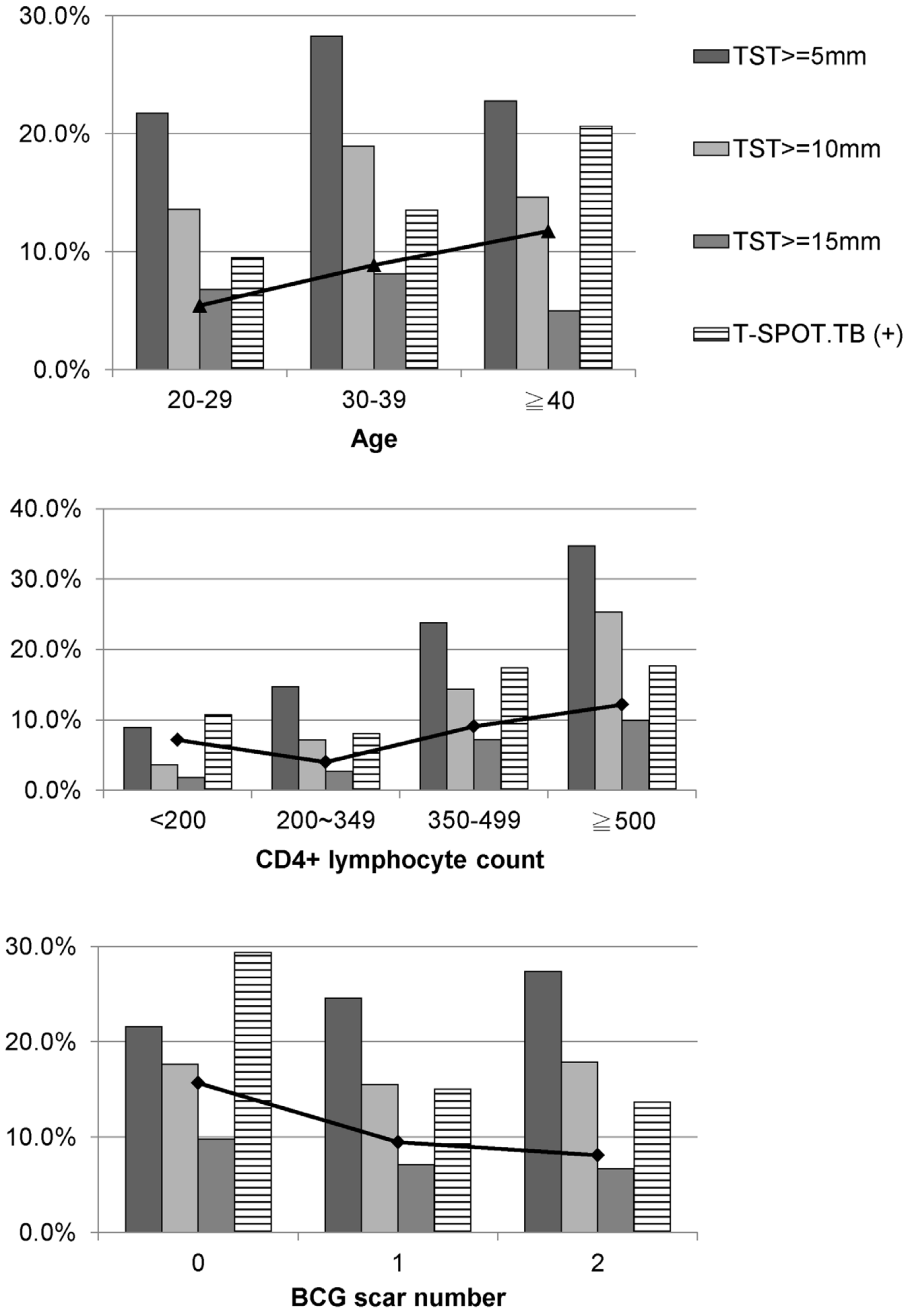
Positive rate of TST by different cut-off point, T-SPOT.TB and dual positive rate. TST =  tuberculin skin test.

By using 10 mm or 15 mm as a cutoff of TST reaction, the agreement was only increased to 82% or 86% with the same kappa value. Figure 2 shows the positive rate of TST response by different cut-off points, T-SPOT.TB and dual positive rate among participants with different age, CD4+ lymphocyte count and BCG scar groups.

### Follow-up and progression to active tuberculosis

After a median follow-up duration of 2.97 years, 5 patients (0.55%) developed active TB were noted and the clinical information was presented in Table 4. All 5 patients had dual positive TST and T-SPOT.TB results at baseline. The incidence of active TB among our participants (excluding patients taking IPT) was 0.17/100 person-year (PY); 95% CI, 0.003–0.29/100 PY. There were another six cases ever been suspected to have TB disease due to abnormal CxR findings during the follow-up period. All cases had further investigations included at least three sets of sputum smear and culture examination. After careful evaluations, all were excluded to have TB disease by the attending physicians.

**Table 4:** Demographic data of the five incident active TB cases.

Case	1	2	3	4	5
IPT	No	No	No	No	Yes
Age (years old)	42.5	42.6	31.8	81.7	26.3
Gender	Male	Male	Male	Male	Male
HIV transmission risk category	IDU	MSM	MSM	Heterosexual contact	MSM
History of TB contact	No	No	Yes	No	No
Prison stay	Yes	Yes	No	No	No
TST size(mm) at enrollment	18	20	16	15	20
CD4+ lymphocyte count at enrollment/TB diagnosis	435/243	130/189	319/314	487/259	578/423
On ART at enrollment/TB diagnosis	No/No	No/Yes	No/No	Yes/Yes	No/No
Interval between enrollment and TB diagnosis (days)	1133	745	360	201	807
TB location	Pulmonary	Pulmonary	Pulmonary	Pulmonary	Pulmonary

*Definition of abbreviations*: IPT = isoniazid preventive therapy; MSM = man who had sex with man; IDU = injection drug use; TST =  tuberculin skin test; ART = highly active antiretroviral therapy.

After exclude individuals with IPT, the TB incidences among HIV-infected individuals were stratified with a positive TST, a positive-SPOT.TB and dual positive results (Table 5). Based on the Poisson regression method, the relative risks for developing active TB in patients with a positive TST, a positive T-SPOT.TB test and dual positive results compared with the risk for patients with negative results were 40-fold, 77-fold and 226-fold, respectively.

**Table 5:** Incidence and relative risks for active TB in HIV-infected individuals with positive TST, T-SPOT.TB and dual positive results, compared with individuals with negative results.

	Total	Cases	Incidence, cases per 100PY (95% CI)	RR*(95% CI)	P value
**All patients**	822	4	0.17 (0.004–0.33)		
**Individuals without IPT**					
** TST**					
TST negative	682	0	-	1	
TST positive	140	4	1.02(0.03–2.02)	40.6(2.1–767.9)	0.014
** T-SPOT.TB**					
negative	737	0	-	1	
positive	85	4	1.72(0.05–3.39)	73.9(3.9–1397.7)	0.004
** Dual Positive of TST and T-SPOT.TB**					
Negative/single positive	790	0	-	1	
Dual positive	32	4	4.93(0.22–9.63)	226.5(12.0–4284)	0.0003

*Definition of abbreviations*: BCG = Bacille Calmette-Guérin; CI = confidence interval; IPT = isoniazid preventive therapy; PY = person-year; TST =  tuberculin skin test. RR =  relative risk.

*Relative risk was calculated by Poisson regression method and the cell with “zero case” was assigned to 0.5 for calculation.

Individuals with IPT were excluded.

**Figure 3:**
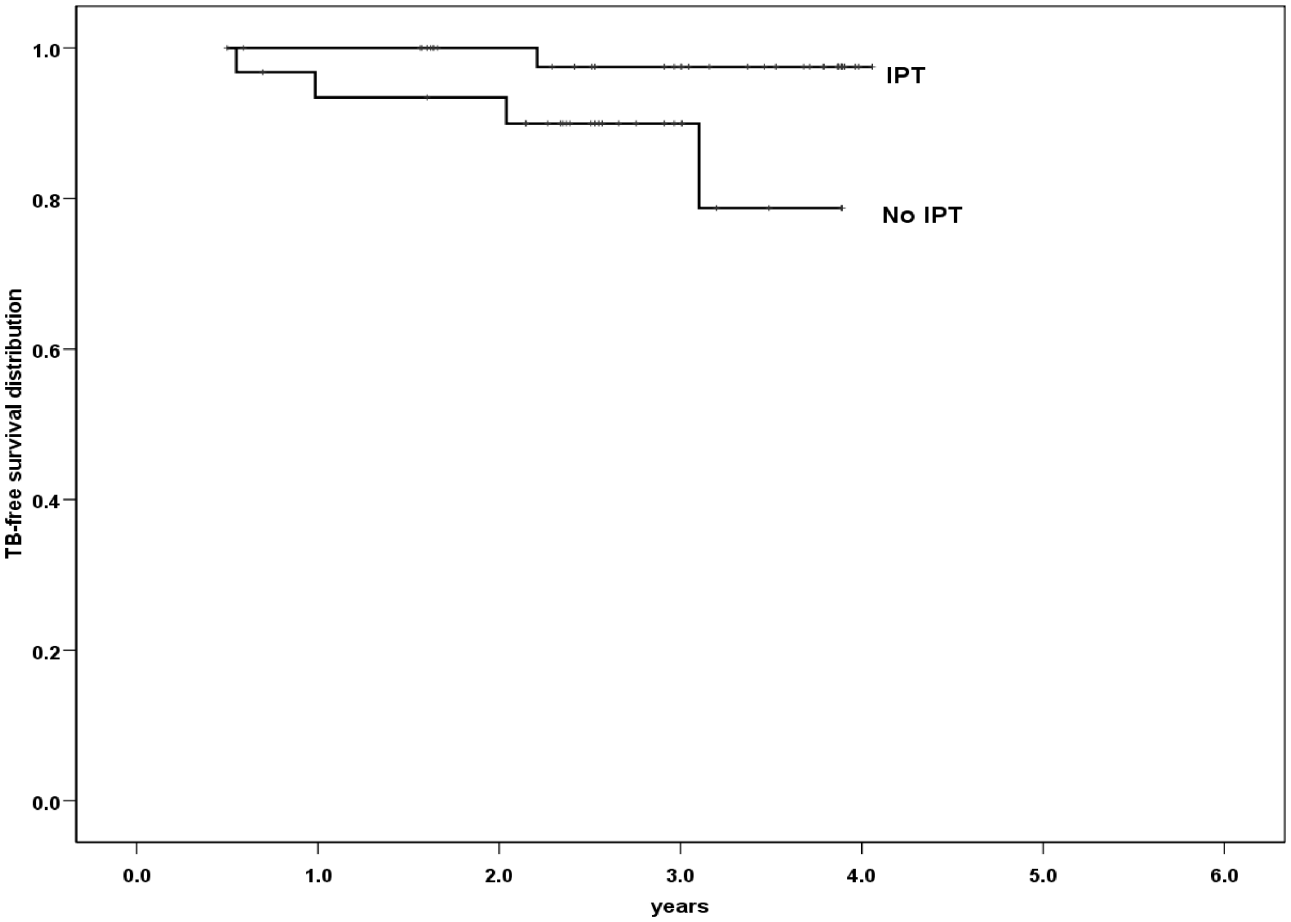
Risk for active TB among patients with dual positive results of TST and T-SPOT.TB stratified by receiving IPT or not. The TB incidence was 4.9 case per 100 PY for participants without IPT vs. 0.66 case per 100 PY for participants received IPT, p<0.05 by log-rank test. TST =  tuberculin skin test; IPT = isoniazid preventive therapy.

There were only 87 (38%) of the 227 TST-positive subjects accepted IPT, with a treatment completion rate of 91.9%. Among patients with dual positive results, there were 4 active TB cases among 32 untreated patients, compared with 1 TB case among 49 patients taking IPT (p<0.05 by the log-rank test, figure 3). Dual positive TST and T-SPOT.TB results were associated with a 7.8-fold increased risk of active TB in patients who did not receive IPT.

### Number needed to treat with different strategies

According to our results, the number needed to treat to prevent one subsequent TB case among patients with a positive TST, a positive T-SPOT.TB and dual positive results was 35, 22 and 8 respectively. If we adopt the strategy that patients with either a positive TST results or a positive T-SPOT.TB test should receive IPT, there will be 30.8% of the enrollee put on treatment. If we adopt that a positive TST or a positive T-SPOT.TB result as criteria, there will be 25% and 14.7% of enrollee put on IPT respectively. If we choose dual positive results as criteria for IPT, then only 8.9% of the enrollee will be put on treatment and this strategy already covers all the active TB patients developed during the follow-up.

## Discussion

In this prospective longitudinal study of LTBI among HIV-infected individuals in Taiwan, we found neither the T-SPOT.TB test nor the TST has high accuracy for the prediction of incident TB. TST had significantly more positive results compared with T-SPOT.TB among BCG-vaccinated HIV-infected individuals except in the participants with CD4+ lymphocyte count <200 cells/mm^3^. Among HIV-infected individuals with dual positive TST and T-SPOT.TB results, after a median 3-year follow up, those received IPT had fewer subsequent TB diseases than participants without IPT. Use dual positive results as the criteria might reduce the number needed to treat for IPT.

The TB incidence among HIV-infected individuals was 0.17 per 100PY, around 2.7 times than general population in Taiwan, which was 62 per 100,000 population in 2008 [11]. Thus the strategy to identify LTBI and provide IPT for HIV-infected population is an important issue in Taiwan. The two tests used in diagnosing LTBI seemed to have lower positive rates in HIV-infected individuals than uninfected population. We compared with the study which enrolled HIV-uninfected inmates revealed that the positive TST rate was 82% with a cut-point of 10 mm and 45% with a cut-point of 15 mm, significantly higher than HIV-infected inmates in our study which was 33% with a cut-point of 5 mm, p<0.001 [13]. HIV-infected inmates also had lower positive IGRA rate compared with uninfected inmates, 18.2% vs. 25%, p<0.001. The lower positive rates may be due to the suppression of immune system. We did found that the CD4+ lymphocyte count played an important role in determining the results of both the TST and the T-SPOT.TB test, and there was a clear trend of decreasing positive rates with decreasing CD4+ lymphocyte counts. The reported influence of the CD4+ lymphocyte count of HIV-infected individuals on the performance of the T-SPOT.TB test seems to be inconsistent. Some studies found no impact [25], [26], [27], but others demonstrated decreasing sensitivity with decreasing CD4+ lymphocyte counts [17], [18], [28]. However, we found in individuals with low CD4+ lymphocyte counts (<200 cells/mm^3^), the T-SPOT.TB test had a higher positive rate than the TST did, different from individuals with higher CD4+ lymphocyte counts. This finding suggested that the T-SPOT.TB test was less affected by CD4+ lymphocyte counts than the TST was in HIV-infected patients. But our sample size was small and more evidence was needed. Furthermore, though some guidelines recommend treat HIV-infected individuals with TST-negative/IGRA-positive results, there were no evidence shown that IPT could improve outcomes on this population [29], [30], [31].

Due to the low specificity, TST may not be a good tool to screen LTBI among BCG-vaccinated HIV-infected individuals, as BCG-vaccinated non-HIV individuals, which is the arduous situation we all faced in Asia [16], [32]. Randomized controlled trials in HIV-infected persons demonstrated that IPT can reduce the risk of active TB in persons with positive TST results, although the BCG vaccination status of the study population was not described [5]. As a result, though the Taiwan's guideline recommended that HIV-infected individuals was stated as the priority for LTBI treatment and recommend using TST as a screening tool, the physicians still hesitated to practice and thus hindered the provision of IPT [22].

One main goal of our study was to develop an appropriate protocol, instead of the current Taiwan's recommendation, to better select HIV-infected individuals for IPT thus to prevent subsequent TB. Unfortunately, there was insufficient evidence to conclude that T-SPOT.TB test was superior to the TST. And the majority of the individuals with positive TST or T-SPOT.TB results did not develop TB. There was only limited number of longitudinal studies focused on the application of IGRA and TST in the LTBI diagnosis among HIV-infected individuals and still no consensus [33], [34]. As to the safety of IPT, potential risks of hepatotoxicity, especially in individuals co-infected with hepatitis virus was also a concern [35]. The high prevalence of hepatitis B virus (HBV) and hepatitis C virus (HCV) infections in HIV-infected population has been demonstrated in many studies, including Taiwan, where 16.4% of HIV-infected individuals had chronic HBV infection and 8.5% had chronic HCV infection [36], [37]. Our study found 11.4% of the 87 participants who received IPT developed acute hepatitis (defined as an increase in serum transaminase level of >3 times the upper limit of normal with symptoms, or an increase in serum transaminase level of >5 times the upper limit of normal without symptoms). Therefore, cautiously selected HIV-infected individuals for IPT to avoid excess adverse events should be taken to consideration. The T-SPOT.TB test can increase the specificity and reduce the impact of BCG vaccination on TST. Number needed to treat for IPT could be reduced significantly by using the dual positive strategy.

Our study has important strengths and limitations. The study involved a prospective large cohort followed up, enabling us to assess the performance of available tests for LTBI diagnosis such as TST and IGRAs by using development of subsequent active TB as the endpoint. In low TB burden areas, such as developed countries, with TB incidences less than 20 per 100,000 person-years, it requires a huge sample size and a prolonged follow-up duration to achieve significant results. Thus there were only a limited number of studies available, especially among HIV-infected patients [34], [38], [39]. On the contrary, study conducted in high TB burden areas with active transmission, such as developing countries with high TB incidences of more than 100 per 100,000 population, infection or reinfection that occurs after baseline LTBI screening might underestimate the negative predictive value of the test results. Taiwan had moderate TB prevalence and over 60% of the incident TB cases raised from endogenous reactivation [40]. Therefore, our study population with low active transmission may be appropriate to estimate the accuracy of the LTBI screening tests. However, the positive rates of both tests were below 30% and only 5 cases developed TB during the follow-up, the sample size may be still small. Besides, our participants had high median CD4+ lymphocyte counts (447 cells/mm^3^), and 40% were on HAART; thus, our results may not be applicable to HIV-infected individuals with lower CD4+ counts or to those who experienced difficulty in obtaining HAART.

In conclusion, adopting positive results of the TST and T-SPOT.TB to screen LTBI among BCG-vaccinated HIV-infected individuals might be feasible. Number needed to treat for isoniazid therapy could be reduced significantly by using the dual positive strategy.
